# Internal Jugular Venous Extension of a Mandibular Osteosarcoma With Literature Review

**DOI:** 10.7759/cureus.19012

**Published:** 2021-10-24

**Authors:** Daniel H Lofgren, Daniel B Hilton, Jens C Brown, Carlos Ramirez

**Affiliations:** 1 Otolaryngology, McLaren Oakland Hospital, Pontiac, USA; 2 Otolaryngology - Head and Neck Surgery, Advanced Ear, Nose & Throat - Head & Neck Surgery, Las Vegas, USA; 3 Otolaryngology - Head and Neck Surgery, Hospital Sisters Health System Sacred Heart Hospital, Eau Claire, USA; 4 Otolaryngology - Head and Neck Surgery, Ascension St. John Hospital, Detroit, USA

**Keywords:** osteosarcoma, intravenous metastasis, head and neck neoplasms, chondroblastic, head and neck reconstruction

## Abstract

Head and neck osteosarcomas (HNOS) account for less than 1% of all head and neck cancers and makeup 6-10% of all primary osteosarcomas. Mandibular osteosarcomas are the second most common subtype of HNOS. They demonstrate higher recurrence rates; however, are amenable to surgery. An 18-year-old male presented with a 2 cm x 3 cm x 2 cm intraoral mass for two months. Biopsy revealed chondroblastic osteosarcoma. Computed tomography revealed extension into the left internal jugular vein. Composite resection of the left mandible, floor of the mouth, ventral tongue, submandibular gland, and modified radical neck dissection with fibular flap repair was performed. Adjuvant chemotherapy and palliative radiotherapy were added. Unfortunately, progressive metastasis to the contralateral mandible and entire spinal cord ensued. We report the first case of head and neck osteosarcoma with intravascular invasion into the internal jugular vein.

## Introduction

Osteosarcoma (OS), the most common primary bone malignancy, generally arises in the long bones of the extremities with a reported incidence varying with age: 4.0 - 5.0 per million per year from 0 to 19 years old [1,2]. It is reported that 6-10% of all OS occurs in the head and neck (HNOS), with a reported incidence of two to three persons per million per year, and typically presents in the fourth decade of life [2-7]. Head and neck osteosarcomas are most commonly found in the skull and facial bones (55-55.6%), followed by the mandible (38.9-45%), with the remaining subsites grouped together (1-5%) including head and neck soft tissues, the parotid gland, nasopharynx, alveolar ridges, glottic larynx, tongue, and laryngeal cartilages [2,8]. Reported local recurrence of HNOS range from 17%-70% vs 5%-7% in extremity OS, and it has been reported that HNOS is less likely to metastasize (10-20%) when compared with long bone disease (53-75%) [2,3,8-11]. It most commonly metastasizes to the lungs but is not known to invade intravascularly [4,6-10]. Cited risk factors for HNOS include prior radiation therapy, Li-Fraumeni syndrome, retinoblastoma, and Rothmund-Thomson syndrome [5,7,9-10,12]. Symptomatic presentation of HNOS varies per tumor size and location leading to palpable masses, local tenderness, dental mobility, paresthesia of the maxillary and mandibular nerves, nasal obstruction, or epistaxis [5,12]

Mandibular Osteosarcoma (MOS), the second most common anatomic location of HNOS, tends to carry the best overall prognosis out of all HNOS subsites, with local recurrence rates of 56%, and a lower likelihood of metastasis to pulmonary and non-pulmonary sites [2-4,7]. In this article, the authors present a patient with MOS and metastatic invasion of the ipsilateral internal jugular vein, which was initially misdiagnosed as jugular thrombosis.

## Case presentation

**Figure 1:**
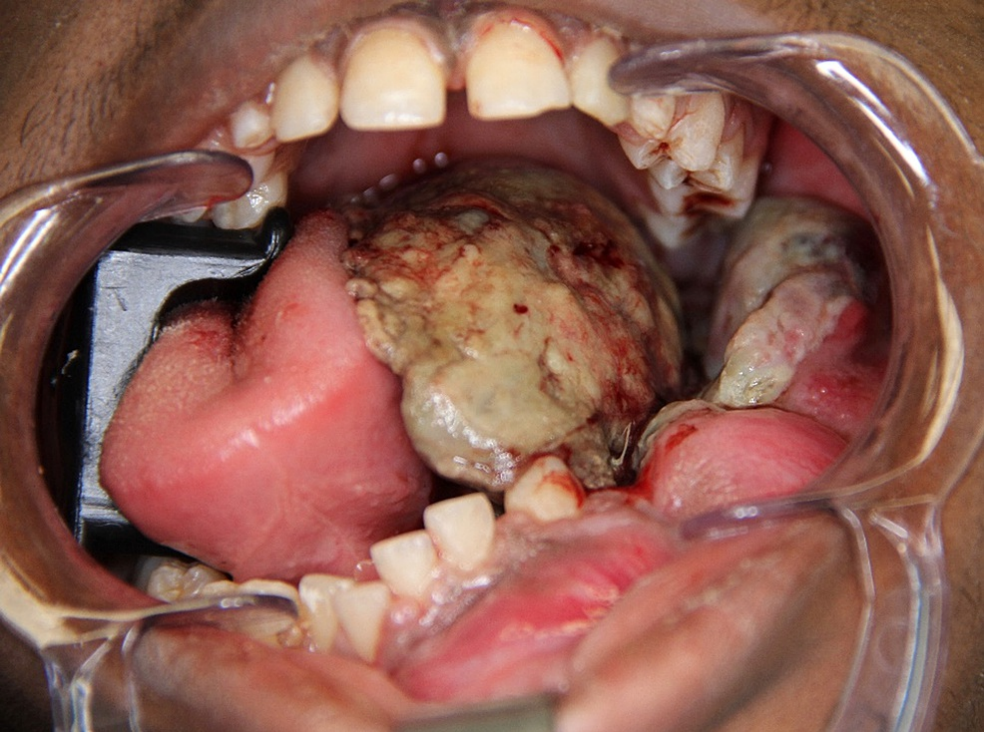
Preoperative evaluation showing the exophytic lesion with submucosal nodularity along the left mandibular body and parasymphysis.

An 18-year-old African American male presented to the oral and maxillofacial surgery clinic with a two-month history of an expansile osteolytic mass of the left mandible, requiring biopsy. Associated symptoms included: localized pain, halitosis, worsening ability to chew solid foods, 10-pound weight loss, night sweats, mouth ulceration, and tooth abnormalities. He denied motor dysfunction of the tongue, dyspnea, dysphagia, odynophagia, or local paresthesia. He denied prior use of tobacco products, had no pertinent family medical history, and showed no improvement after a short course of oral amoxicillin. Per the patient, from the time of initial biopsy to the authors' evaluation, the lesion increased dramatically in size. Oral exam revealed a 2 cm x 3 cm x 2 cm exophytic mass emanating inferior to teeth 18 and 19 with extension to left parasymphysis and multiple submucosal nodules along the buccal and lingual aspect of the left mandibular body and parasymphysis as seen in Figure 1. Teeth numbers 18 through 25 were grossly mobile. The patient had normal facial movement, with some resting facial asymmetry secondary to the mass, and lacked objective tenderness or regional paresthesia. All cranial nerves were intact, and he had no neck masses or lymphadenopathy.

**Figure 2:**
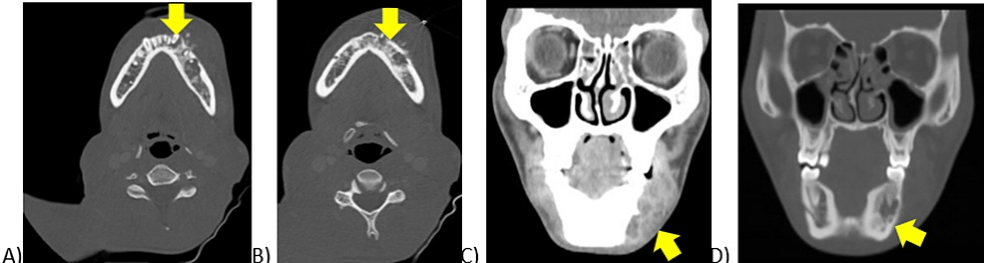
Axial (A and B) and coronal (C and D) non-contrast maxillofacial CT showing involvement of the left mandibular parasymphysis and surrounding tissues.

**Figure 3:**
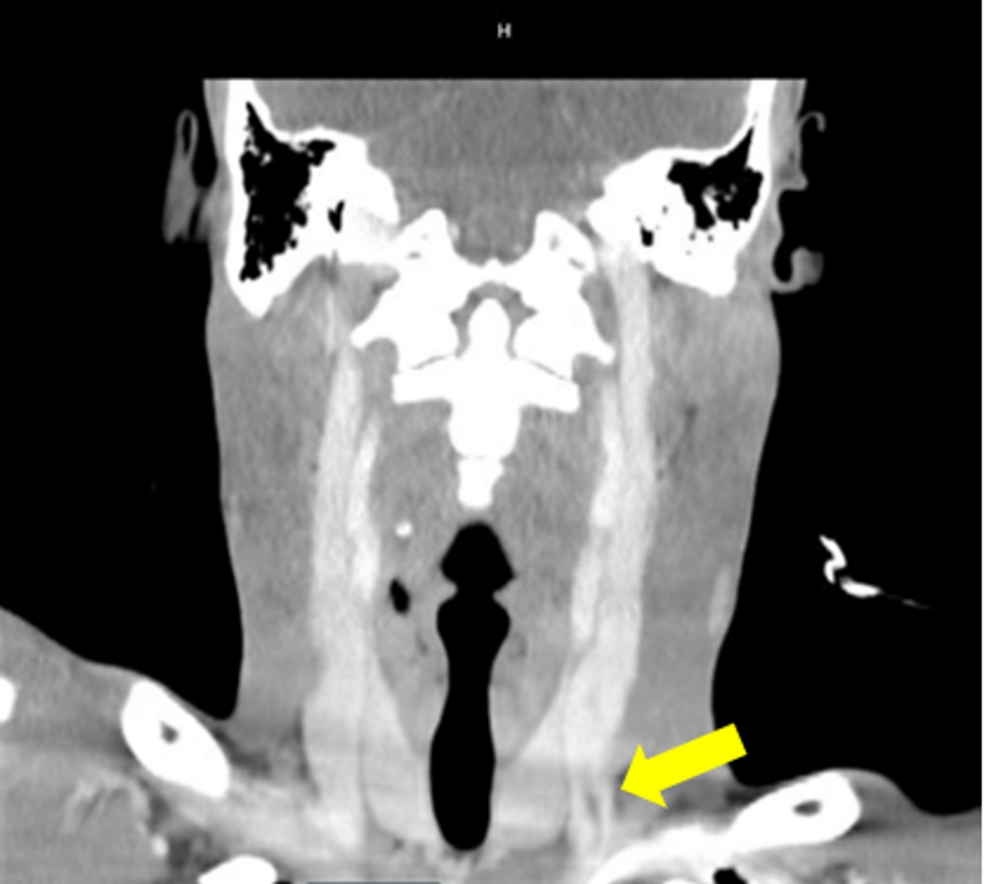
Contrasted CT scan of the neck in the coronal plane showing a tubular density in the left internal jugular vein.

**Figure 4:**
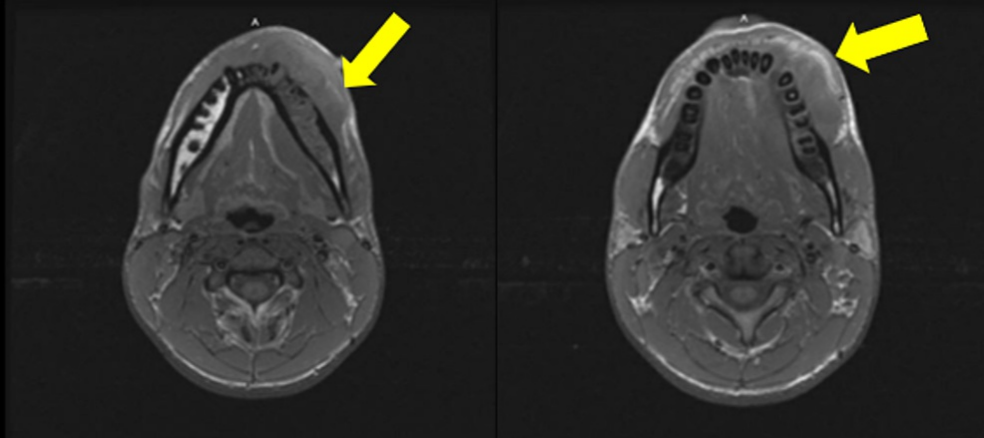
Axial cut T1 MRI showing adjacent soft tissue involvement of left mandibular parasymphysis.

**Figure 5:**
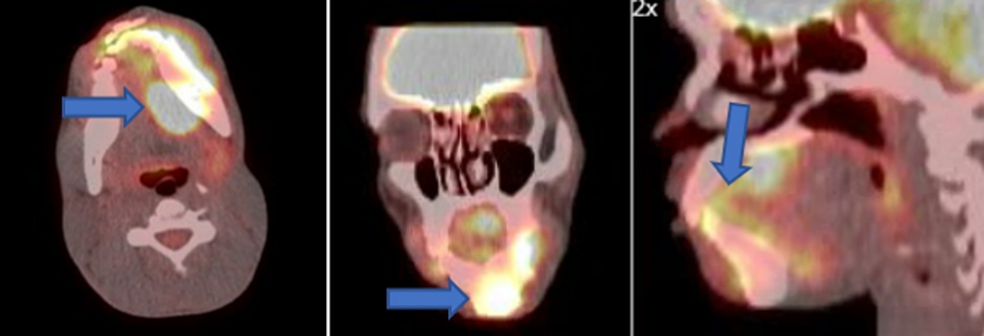
Preoperative PET CT scan showing localization of the tumor bed within the left mandible, floor of mouth, tongue, and submandibular gland.

Subsequent pathology was consistent with chondroblastic osteosarcoma and immunohistochemistry of the sample was positive for CD99 and vimentin, but negative for schwannian-100 (S100), anti-muscle actin antibody (HHF35), cytokeratin, and epithelial membrane antigen (EMA). A computed tomography scan (CT), seen in Figure 2, revealed an aggressive left mandibular lesion with prominent soft tissue invasion and scattered nonspecific cervical chain lymph nodes. The CT also revealed a tubular, hypodense lesion anterior and extending into the left jugular vein, which was reported as a deep venous thrombosis (DVT) seen in Figure 3. Magnetic resonance imaging (MRI) demonstrated the mass diffusely involving the left mandible from the angle to the mental protuberance, crossing midline, and involving the right mental protuberance (Figure 4). An associated enhancing soft tissue mass was seen extending into the surrounding soft tissue structures, most pronounced involving the sublingual space, and resulted in the displacement of the adjacent tongue musculature. A positron emission tomography (PET) CT scan demonstrated intense metabolic activity in soft tissue adjacent to the left mandibular body as described on prior CT/MRI without lymphatic spread or distant metastasis noted in Figure 5.

**Figure 6:**
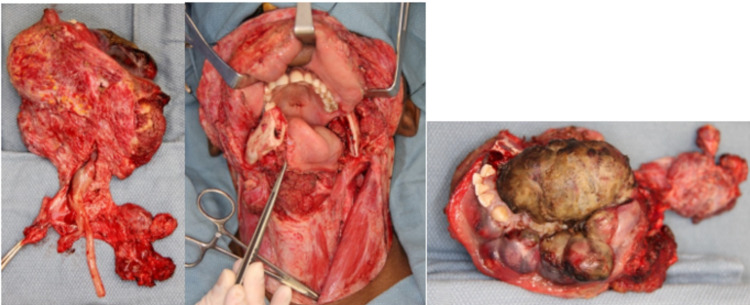
Surgical resection of left mandible, floor of mouth, ventral tongue, and submandibular gland with a modified radical neck dissection.

Three weeks later, the patient presented for resection of the tumor, which was noted to have doubled in size since surgical planning. He underwent composite resection of the mandible from right parasymphysis to left ramus, the floor of the mouth, ventral tongue, submandibular gland, left internal jugular vein, and modified radical neck dissection, sparing the spinal accessory nerve and sternocleidomastoid muscle, shown in Figure 6. Although a neck dissection is not standard of care in OS, it was completed because of visible invasion of disease within the surrounding musculature and the vasculature including the facial artery and vein. Gross invasion of tumor into left internal jugular vein was evident during dissection with nests of tumor extension into the mediastinal vessels, which was confirmed by histopathology. The defect was repaired with an osteomyocutaneous fibula free flap with a custom reconstruction plate and a left thigh split-thickness skin graft placed along the buccal mucosa. Permanent surgical pathology confirmed the 7.5 cm mass as a poorly differentiated chondroblastic osteosarcoma with a positive anterior bony margin of the mandible involving the sub-dermis of the mental region. Of the lymph nodes taken, zero of the 38 were positive for metastasis. There was no invasion of the submandibular gland, but there were microscopic nests of local invasion into the left anterior digastric muscle. 

**Figure 7:**
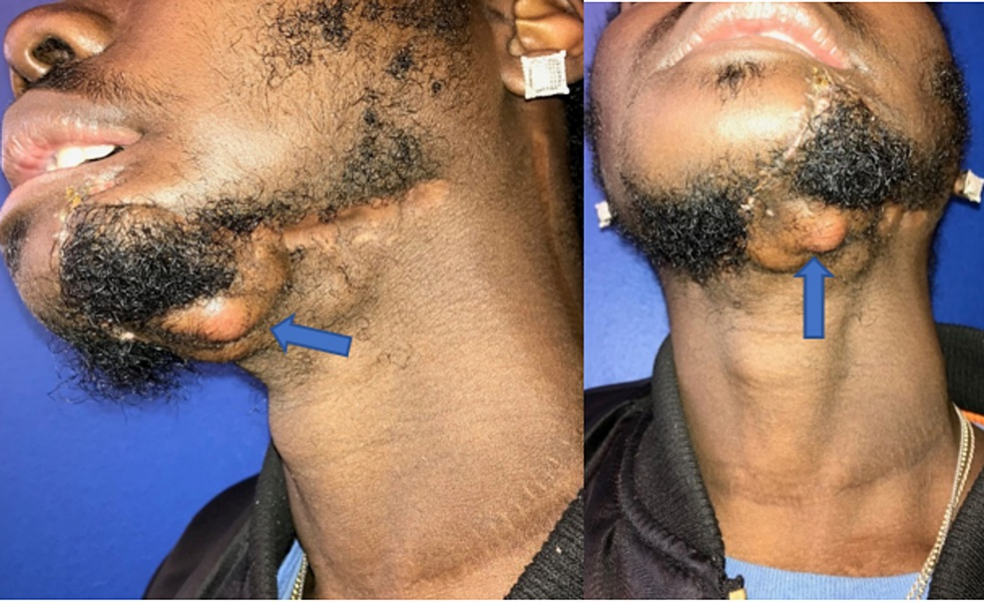
Initial recurrence months after composite resection.

Three weeks later, he began chemotherapy following the Children’s Oncology Group protocol AOST0331, which consisted of 29 weeks of methotrexate, cisplatin, and adriamycin. As seen commonly with both radiotherapy and chemotherapy, obliteration of microvasculature delayed angiogenesis, and local tissue fibrosis can lead to reconstructive complications, so after extended tumor-board discussion, adjuvant radiotherapy was not considered at that time. Approximately three months into his chemotherapy treatment, the patient began to note superficial exposure over his left hemi-mandible, leading to significant oral-cutaneous communication as seen in Figure 7. The left-sided hardware was removed along with the left mandibular fistula and corresponding skin track, and followed by a right deep segmental mandibulectomy to eradicate any potential residual disease. This entire defect was reconstructed with a right osteomyofascial fibular free flap and microvascular anastomosis. Surgical pathology of the right mandibular segmental resection showed a 2.5 millimeter (mm) poorly differentiated chondroblastic osteosarcoma with gross local submental vessel invasion. After tumor-board discussion with oncology, the patient was considered a poor responder to chemotherapy due to vascular invasion and tumor growth despite 16 weeks of chemotherapy; however, he elected for completion of his 29 weeks treatment regimen.

**Figure 8:**
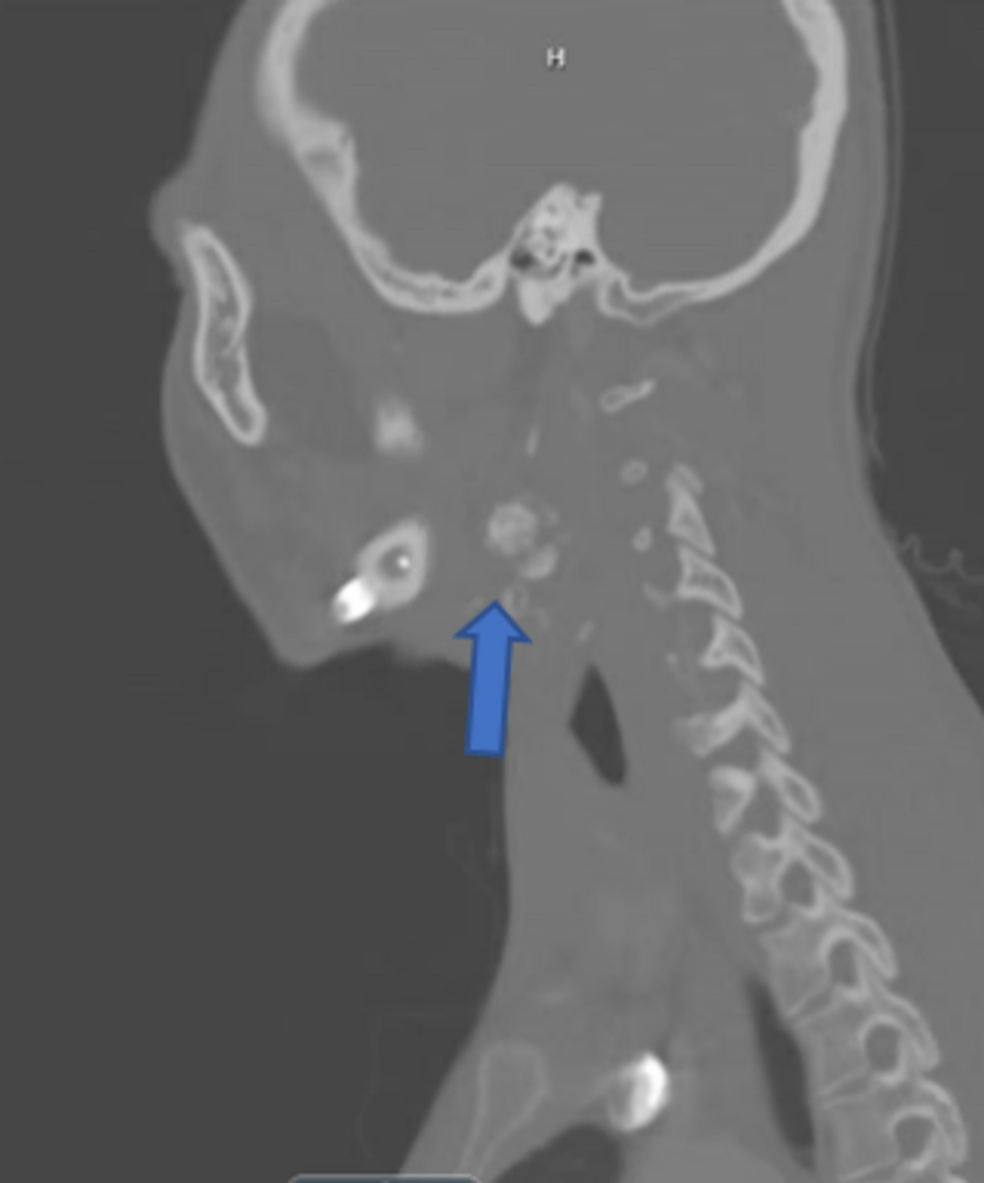
Non-contrasted CT scan, sagittal cut, showing recurrence along with cervical spine metastasis.

**Figure 9:**
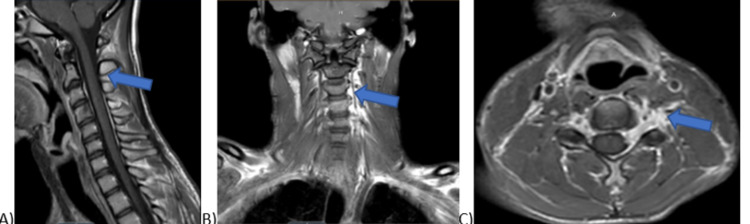
Repeat T1 MRI with Gadolinium contrast showing involvement of left C4-C6 nerve roots and encasement of the vertebral artery in sagittal (A), coronal (B), and axial (C) view respectively.

Approximately eight months later the patient developed left upper extremity weakness and left-sided lymphadenopathy. A left-sided parasymphyseal orocutaneous fistula had developed with exposed hardware. CT (Figure 8) and MRI (Figure 9) of the neck revealed marginal recurrence, left levels I and II metastatic lymph nodes that encased the C4-6 nerve roots and left vertebral artery, and osseous metastasis to the C4 vertebral body. A CT of the chest did not reveal any pulmonary metastasis. Open lymph node biopsy of the affected submental lymph nodes was positive for tumor invasion. The orocutaneous fistula was then excised and repaired by local tissue rearrangement.

**Figure 10:**
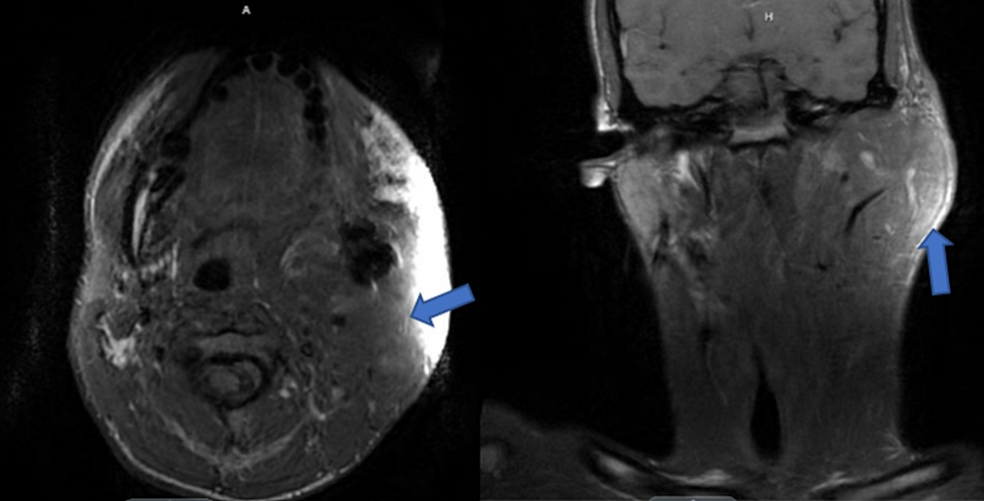
T1 MRI without contrast revealing diffuse recurrence and infiltration of pterygoid muscles, parapharyngeal space, parotid space, masseter, and left temporalis on axial and coronal views respectively.

He elected for external beam radiotherapy for cervical metastasis, which resulted in some improvement in left upper extremity function. Hospice was discussed at this time but was declined by the patient and his mother. Denosumab and sorafenib were initiated for palliative therapy, but the progression of local disease proceeded, and the biologics were stopped due to worsening side effects. A follow-up MRI (Figure 10) revealed disease infiltration of the left temporalis, masseter, lateral pterygoid, parapharyngeal space, parotid space, posterior triangle, and lateral to the previously resected mandible.

**Figure 11:**
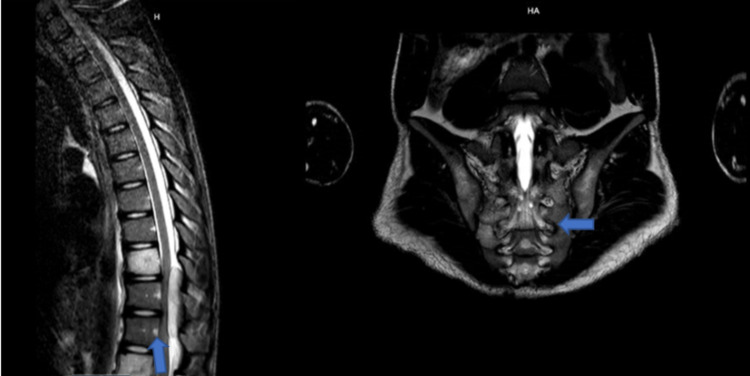
Non-contrasted T2 MRI of the thoracic spine, lumbar spine, and pelvis showing diffuse metastasis on sagittal and axial cut respectively.

One month later the patient reported to the emergency room due to worsening paresthesia of bilateral lower extremities and MRI revealed extensive bony metastases involving the entire sacrum and bilateral ilium with right S1-S3 neural foramina tumor extension and posterior epidural invasion at T10-L1 producing spinal stenosis and cord compression (Figure 11). Neurosurgery was unable to offer surgical treatment and recommended external beam radiation for palliative treatment. The patient underwent another round of radiotherapy to the spinal cord for pain control, which temporized his symptoms, but he was lost to follow up afterward. The care team was later notified of his death 22 months after the initial diagnosis.

## Discussion

Clear surgical margins have been reported to be the most significant positive prognostic factor for overall survival, local control, and disease-free survival in MOS [2,11,12]. Other reported positive factors include presentation within the symphyseal region, which showed a 100% five-year overall survival rate, and the use of adjuvant chemotherapy [6,7,11]. Age greater than 60 years old, involvement of the bony condyle, and positive margin status demonstrated poor prognosis [6,7,11].

Historically, surgical resection of HNOS with wide margins is the mainstay of treatment, but the anatomic complexity of the head and neck can prevent adequate margins [2,5,13-15]. A National Cancer Database Report reported a five-year disease-specific survival rate of 59.7% for all HNOS; however, removal of non-surgically treated patients increased the five-year disease-specific survival to greater than 70%. Patients who underwent non-surgical treatment only had a five-year survival of 21.7%. Those with regional or distant diseases had a five-year survival rate of 42% [2]. Seikaly et. al. reported a two-year and five-year overall survival rate in Canadian patients of 79%+/-7% and 57%+/-9%, respectively [11].

Highlighted in the case above, the patient did benefit symptomatically from palliative radiotherapy, but it is unlikely that adjuvant radiotherapy after his composite resection would have prevented the further distant progression of his disease. The patient in the case above initially presented with a histopathologically confirmed intravascular invasion of the internal jugular vein (IJV), suggesting a more severely advanced lesion at the onset, likely leading to his poor outcome. Overall, evidence of adjunct radiotherapy in HNOS treatment is lacking. Thariat and colleagues failed to demonstrate the benefit of radiotherapy in HNOS but noted a small sample size as a limiting factor of the study [12]. Another study, of HNOS patients with positive or uncertain margins, compared surgery with and without radiation, and reported a statistically significant improvement in local control, overall survival, and disease-free survival in the combination surgery and radiation group [7,8]. Chen et al. also found significantly improved local control in HNOS lesions treated with post-surgical radiotherapy regardless of other co-factors [6].

Both adjuvant and neoadjuvant chemotherapy are other HNOS treatment options that lack high-grade evidence [3,9-11,13,15,16]. Recent literature supports multimodality therapy for intermediate to high-grade lesions for local control, which is associated with decreased distant metastasis, and improvement in overall and disease-specific survival [2,5-6,8-9-11,13,17]. Mucke et al. noted improvement in two & five-year overall survival rates with the addition of neoadjuvant chemotherapy (100% and 66.7% versus 66.7% and 41.7%) [4]. Two studies showed comparable five-year survival rates and decreased local recurrence suggesting neoadjuvant chemotherapy increases survival in HNOS with poor prognostic factors [6,10]. The optimal timing of chemotherapy has not been well defined, although theoretically, neoadjuvant treatment shrinks pre-surgical tumor bulk leading to conservative surgical margins and improved survival [13]. For the practicing head and neck surgeon, resection will still need to start at the pre-treatment margins.

Lastly, within the literature, there have been a handful of case reports describing intravascular metastasis of osteosarcoma, but none involving HNOS. One noted case study described intravascular embolic metastases to pulmonary vasculature from primary OS lesion in his right femur [18,19]. Another case report noted inferior vena cava extension of a local pelvic OS [20]. After searching the PubMed, Cochrane Review, and Evidence for Policy and Practice Information and Co-ordinating Centre (EPPI-Centre) databases, the authors conclude that this is the first reported case of gross intravascular tumor extension by an already rare mandibular osteosarcoma.

## Conclusions

After an extensive literature review, no reported cases were discovered reporting HNOS with an intravascular extension of tumor into any major blood vessels. Early identification and surgical excision with wide margins are the most viable treatment in MOS, but with early vascular invasion, excision alone is not always feasible. There appears to be a role for radiotherapy and chemotherapy, but unfortunately, it is not well defined in HNOS literature. The authors hope that this report could provide insight to providers on the identification and treatment of intravascular HNOS invasion and prevent further morbidity and mortality in this patient population.
